# Bcl6 controls the stability and suppressive function of regulatory T cells in head and neck squamous cell carcinoma

**DOI:** 10.1016/j.gendis.2024.101505

**Published:** 2024-12-26

**Authors:** Shuqiong Wen, Xingxing Su, Junyi Guo, Zhanpeng Ou, Lisha Wang, Zhengliang Yue, Jing Zhao, Ling Ran, Jianjun Hu, Yuzhu Wang, Mengqu Ran, Qinyi He, Ping Ji, Lilin Ye, Zhiyu Chen, Lifan Xu, Qizhao Huang

**Affiliations:** a Chongqing Key Laboratory of Oral Diseases, Chongqing Municipal Key Laboratory of Oral Biomedical Engineering of Higher Education, The Affiliated Stomatological Hospital of Chongqing Medical University, Chongqing 401147, China; b Guanghua School of Stomatology, Guangdong Provincial Key Laboratory of Stomatology, Stomatological Hospital, Sun Yat-Sen University, Guangzhou, Guangdong 510055, China; c Department of Hepatobiliary Surgery, Southwest Hospital, Third Military Medical University, Chongqing 400038, China; d Department of Stomatology, Third Xiangya Hospital, Central South University, Changsha, Hunan 410013, China; e Institute of Immunology, Third Military Medical University, Chongqing 400038, China; f Biomedical Analysis Center, Third Military Medical University, Chongqing 400038, China; g Institute of Immunological Innovation and Translation, Chongqing Medical University, Chongqing 400016, China

**Keywords:** Anti-tumor immunity, B-cell lymphoma 6 (Bcl6), FX1, Head and neck squamous cell carcinoma (HNSCC), Immune checkpoint blockade, Treg cells

## Abstract

Head and neck squamous cell carcinoma (HNSCC) ranks as the sixth most common cancer globally. Most studies in HNSCC demonstrated that regulatory T (Treg) cells confine the anti-tumor activity of effector T cells which may contribute to the immune escape and uncontrolled tumor progression. Here, we uncovered that the specific abrogation of Bcl6 in Treg cells resulted in significantly delayed malignant transformation of 4NQO-induced tumorigenesis. Bcl6 deficiency impairs the lineage stability of Treg cells by down-regulating the histone H3K4 trimethylation. Importantly, Bcl6 inhibition repressed the tumor growth of murine HNSCC and exhibited synergistic effects with immune checkpoint blockade therapy. These findings suggest that Bcl6 can be exploited as a promising therapeutic target for HNSCC treatment.

## Introduction

According to the Global Cancer Statistical Report in 2022, nearly one million people worldwide were diagnosed with head and neck cancer annually, among which head and neck squamous cell carcinoma (HNSCC) accounts for about 90% of all head and neck cancers.^1^^,^^2^ In recent years, the incidence of HNSCC has been rising quickly while the overall prognosis is poor, with a 5-year survival rate of less than 50%. Despite the comprehensive treatment approaches, including surgery, radiation, and chemotherapy, HNSCC is prone to recurrence and metastasis. Currently, effective treatments for advanced or recurrent HNSCC are still being investigated.^3^ Therefore, a better understanding of the underlying mechanisms driving tumorigenesis and progression is crucial to develop new treatment strategies for HNSCC.

Currently, cancer immunotherapy especially the immune checkpoint blockade (ICB) therapy, such as monoclonal antibodies (mAbs) against programmed death-1 receptor (PD-1) and its ligand (PD-L1), and cytotoxic T lymphocyte-associated antigen 4 (CTLA4), have shown promising therapeutic efficacy in various types of cancer. Pembrolizumab was the first ICB antibody approved for treating metastatic or recurrent HNSCC.^4^^,^^5^ However, only a minority of HNSCC patients benefit from ICB therapy, with a response rate below 20% which is largely attributed to the immunosuppressive tumor microenvironment.^6^^,^^7^ Regulatory T (Treg) cells, the major subtype of the immunosuppressive cells in the tumor microenvironment, represent 10%–50% of tumor-infiltrating CD4^+^ T cells while only occupying 2%–5% of peripheral CD4^+^ T cells in healthy individuals.^8^ For several types of solid tumors, for instance, hepatocellular carcinoma, non-small cell lung cancer,^9^ renal cell carcinoma,^10^ and HNSCC,^11^ increased intra-tumoral Treg cells and Treg/CD8^+^ T cell ratio have been shown to be associated with poorer clinical outcome. However, the potential mechanisms regulating Treg cells during tumorigenesis remain obscure.

B-cell lymphoma 6 (Bcl6) is a master transcriptional repressor highly conserved between mice and humans. It is a lineage marker of T follicular helper cells and plays a crucial role in the generation of germinal centers.^12^ Previous studies have shown that Treg cells lacking Bcl6 overexpress GATA3 (GATA binding protein 3), a Th2-determining transcription factor, thereby aggravating Th2-type airway inflammation.^13^^,^^14^ We previously found that the kinase mTORC1 (mechanistic target of rapamycin complex 1) promoted the differentiation of T follicular regulatory cells (a subset of Treg cells) through TCF1 (T cell factor 1)/Bcl6 axis in response to protein immunization or viral infection.^15^ High levels of Bcl6 in Treg cells may correlate with poor overall survival of colorectal cancer patients and increased risk of lymph node metastasis of melanoma patients.^16^ Nevertheless, whether and how Bcl6 regulates the Treg cells in HNSCC needs to be further elucidated.

In this project, we found that the deficiency of Bcl6 in Treg cells impaired the lineage stability and suppressive function of Treg cells, thus promoting the priming and activation of anti-tumor immune responses which led to significantly delayed malignant transformation in 4NQO-induced HNSCC model. Moreover, Bcl6 inhibitor FX1 repressed the tumor progression of subcutaneous HNSCC and exhibited a synergized effect with PD-1/PDL-1 ICB.

## Materials and methods

### Mice

*Bcl6*^*flox/flox*^ and *Foxp3*^*YFP-Cre*^ knock-in mice within the C57BL/6 background were purchased from Jackson Laboratory. They were bred with each other to generate *Bcl6*^*flox/flox*^ *Foxp3*^*Cre*^ mice (knock-out/KO mice) and *Bcl6*^*flox/flox*^ mice were used as wild-type (WT) controls. Mice were age-matched within experiments, and both males and females were included without randomization or “blinding”. C3H mice were purchased from GemPharmatech. All animal studies were conducted in accordance with the guidelines of the Institutional Animal Care of Sun Yat-Sen University and Use Committee procedures (approval number: SYSU-IACUC-2022-000696).

### 4NQO-induced carcinogenesis model

4-nitroquinoline-1-oxide (4NQO, Sigma–Aldrich) was delivered to 8–12-week-old KO and WT mice in the drinking water at a concentration of 100 μg/mL for consecutive 16 weeks, followed by 6–8 weeks of sterile water only as previously described.^17^^,^^18^ The 4NQO water was replaced weekly.

### Inoculated HNSCC tumor models

C3H mice were subcutaneously inoculated with 1 × 10^6^ SCC7 cells. When tumors became palpable (day 5), SCC7-implanted mice were randomly divided into four treatment groups: i) control; ii) anti-PDL1 antibody; iii) Bcl6 inhibitor FX1, and iv) combination therapy of FX1 and anti-PDL1. Mice in the FX1 group and combination group were intraperitoneally injected with 70 mg/kg FX1 (Selleck Chemicals) daily from day 5 for consecutive 8 days according to the preliminary experiment (data not shown). Mice in the anti-PDL1 group and combination group were intraperitoneally treated with 150 μg anti-PDL1 (Bio X Cell) according to the previous report^19^ at days 6, 8, 10, and 12. Tumors were measured daily with calipers starting from day 5, and tumor volumes were calculated using the formula: volume (mm^3^) = ((width)^2^ × length) ÷ 2.

### Histopathological analysis

Oral lesions were identified and photographed. Next, mice were euthanized, and tongues were harvested and longitudinally bisected. One-half of each tongue tissue was fixed in 10 % neutral phosphate-buffered formalin for 24 h and then embedded in paraffin. The embedded sections (4 μm) were stained with hematoxylin and eosin within the sequential steps of deparaffinization, staining, dehydration, clearing, and coverslipping as previously described^20^ and these stained slides were scanned by Olympus OlyVIA. Histopathological analysis was blindly performed by two certified oral pathologists according to the pathological grading criteria of head and neck tumors described by the WHO.^21^

### Tissue dissociation and single-cell suspension preparation

Draining lymph nodes (dLNs) were extracted and mechanically disrupted using super-frosted microscope slides. The second part of the bisected tongue tissue and SCC7 tumors were cut into several pieces and digested in 1 mg/mL collagenase IV (Gibco) and 0.1 mg/mL DNase Ⅰ (Sigma–Aldrich) solution, and dissociated using GentleMACS dissociator (Miltenyi Biotec). All cell suspension was filtered by 70 μm cell strainers before staining.

### Flow cytometry analysis

Cell surface staining was performed in phosphate buffer saline with 2% fetal bovine serum (Gibco) at 4 °C for 30 min. Then, cells were washed three times to remove excess surface antibodies. The staining of nuclear targets and H3K4 methylation were performed using the Foxp3/Transcription Factor Staining Buffer Set (00–5523; eBioscience); primary unconjugated antibodies to H3K4me1 and H3K4me3 were detected by secondary staining with anti-rabbit IgG Alexa Fluor® 488 antibody (A21206; Invitrogen). For the detection of cytokine production, cells were stimulated *in vitro* with 50 ng/mL PMA (Sigma–Aldrich) and 1 μg/mL ionomycin (Sigma–Aldrich) in the presence of Golgi Plug (BD Biosciences) and Golgi Stop (BD Biosciences) at 37 °C and 5% CO_2_ for 5 h. Next, cells were stained with cytokine antibodies with the Cytofix/Cytoperm Fixation/Permeabilization Kit (554722; BD Biosciences). Data were acquired by LSRFortessa cytometer (BD Biosciences) and analyzed using FlowJo software (Tree Star). All antibodies used for flow cytometry are listed in Table S1.

### mRNA sequencing and analysis

The dLN CD4^+^ CD25^+^ GITR^+^ Treg cells from mice at week 24 of the 4NQO-induced carcinogenesis model were sorted by FACSAria II (BD Biosciences). The total RNA of the sorted cells was extracted using RNeasy Mini Kit (163040872; QIAGEN) for total RNA sequencing. Differentially expressed genes were identified within the cutoff criteria of |log_2_ fold change| >0.5 and false discovery rate <0.05 using the DESeq2 package in R (V.1.30.0). The selected genes were visualized by R package pheatmap (V.1.0.12). Differentially expressed genes were used for gene ontology functional enrichment analysis by R package clusterProfiler (V.3.18.0).

### Quantitative reverse transcription PCR analysis

To confirm the RNA sequencing results, cDNA was generated from isolated RNA with the RevertAid H Minus First Strand cDNA Synthesis Kit (ThermoFisher Scientific). Quantitative reverse transcription PCR was performed using the QuantiNova SYBR Green PCR Kit (Qiagen) on a CFX96 Touch Real-Time System (Bio-Rad). All primer sequences are listed in Table S2.

### *In vitro* suppression assay

The suppressive capacity of Treg cells was detected as previously described.^22^ Treg cells were sorted from the dLNs of 4NQO-treated KO and WT mice with fluorescence-activated cell sorting. Splenic CD8^+^ T cells were obtained from naïve WT mice using a CD8^+^ T cell isolation kit (Miltenyi Biotec) and were labeled with CellTrace™ Violet Cell Proliferation Kit according to the manufacturer's instructions (65–0842; eBioscience). Various numbers of Treg cells (at the indicated Treg:CD8 ratios) were cultured with preceding 3 × 10^4^ labeled CD8^+^ T cells in the presence of anti-CD3 (0.5 μg/mL; BD Biosciences) and anti-CD28 (0.5 μg/mL; BD Biosciences). Cell division was evaluated after 3 days of culture by flow cytometry.

### Statistical analysis

All statistical analyses were performed by Prism software (GraphPad). An unpaired two-sided student's *t*-test was used for between-group comparisons. Fisher exact test was used to compare the proportion of tongue lesions. Tumor volumes at different time points were calculated by two-way ANOVA with a Turkey post hoc test for comparison among groups. Other experiments comparing more than two groups were evaluated by one-way ANOVA. *P* < 0.05 was considered statistically significant (∗*P* < 0.05, ∗∗*P* < 0.01, ∗∗∗*P* < 0.001, and ∗∗∗∗*P* < 0.0001). *P* values > 0.05 represented no significance.

## Results

### Bcl6 deficiency in Treg cells results in delayed malignant transformation in 4NQO-induced tumorigenesis model

**Figure 1:**
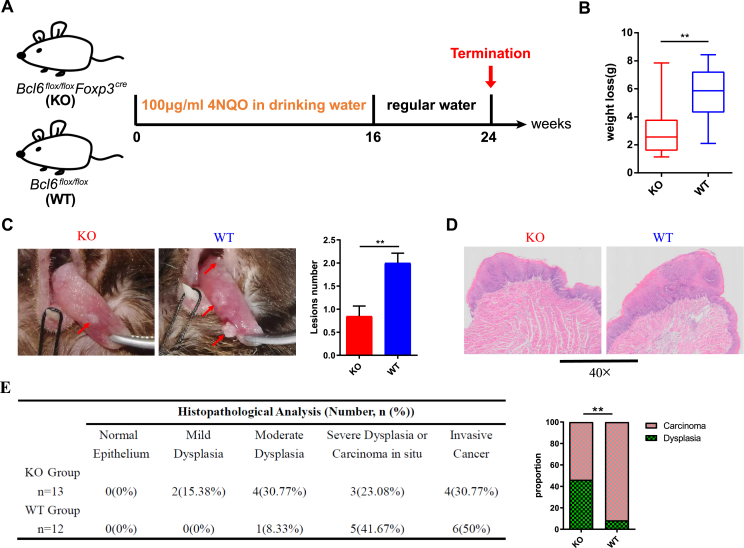
Bcl6 deficiency in Treg cells results in delayed malignant transformation in the 4NQO-induced HNSCC model. **(A)** The schematic diagram of the 4NQO-induced HNSCC model. Drinking water containing 4NQO was administrated to KO mice (*n* = 13) and WT mice (*n* = 12) weekly for consecutive 16 weeks and then changed to regular water until the endpoint of week 24. **(B)** Weight loss was calculated by the body weight at week 16 subtracted from that at week 24. **(C)** Representative images of the tongues in different groups. Red arrows indicate the white lesions. **(D)** Representative hematoxylin and eosin staining of the tongue lesions. **(E)** The histological proportion of tongue lesions. Data were presented as mean ± standard error of the mean calculated by unpaired *t*-tests for (B, C) and the Kruskal–Wallis test for (E); *n* ≥ 12; data are representative of at least three independent experiments; ∗∗*P* < 0.01.

To elucidate the role of Bcl6 in Treg cell's immune response in HNSCC, we established the 4NQO-induced HNSCC model in *Bcl6*^*flox/flox*^ *Foxp3*^*Cre*^ (KO) mice and littermate *Bcl6*^*flox/flox*^ (WT) mice. Mice were given sterile water containing 4NQO at a concentration of 100 μg/mL for 16 consecutive weeks, followed by sterile water for 8 weeks (Fig. 1A). At the endpoint of week 24, KO mice suffered less weight loss compared with WT mice (Fig. 1B). Correspondingly, KO mice exhibited smaller and fewer tongue lesions compared with WT counterparts (Fig. 1C). These alterations were further corroborated by histological examination with hematoxylin and eosin staining. As expected, WT mice revealed larger lesions compared with KO mice (Fig. 1D). The lesions in most WT mice (11/12 mice, 91.67 %) progressed into carcinoma *in situ* or even invasive carcinoma while the lesions in nearly half of KO mice (6/13 mice, 46.15 %) were still under the dysplasia stage (Fig. 1E). Collectively, these data suggested that Bcl6 deficiency in Treg cells could effectively delay the malignant transformation of HNSCC.

### Bcl6 deficiency in Treg cells augments T-cell responses in both tongue immune microenvironment and tumor dLNs

**Figure 2:**
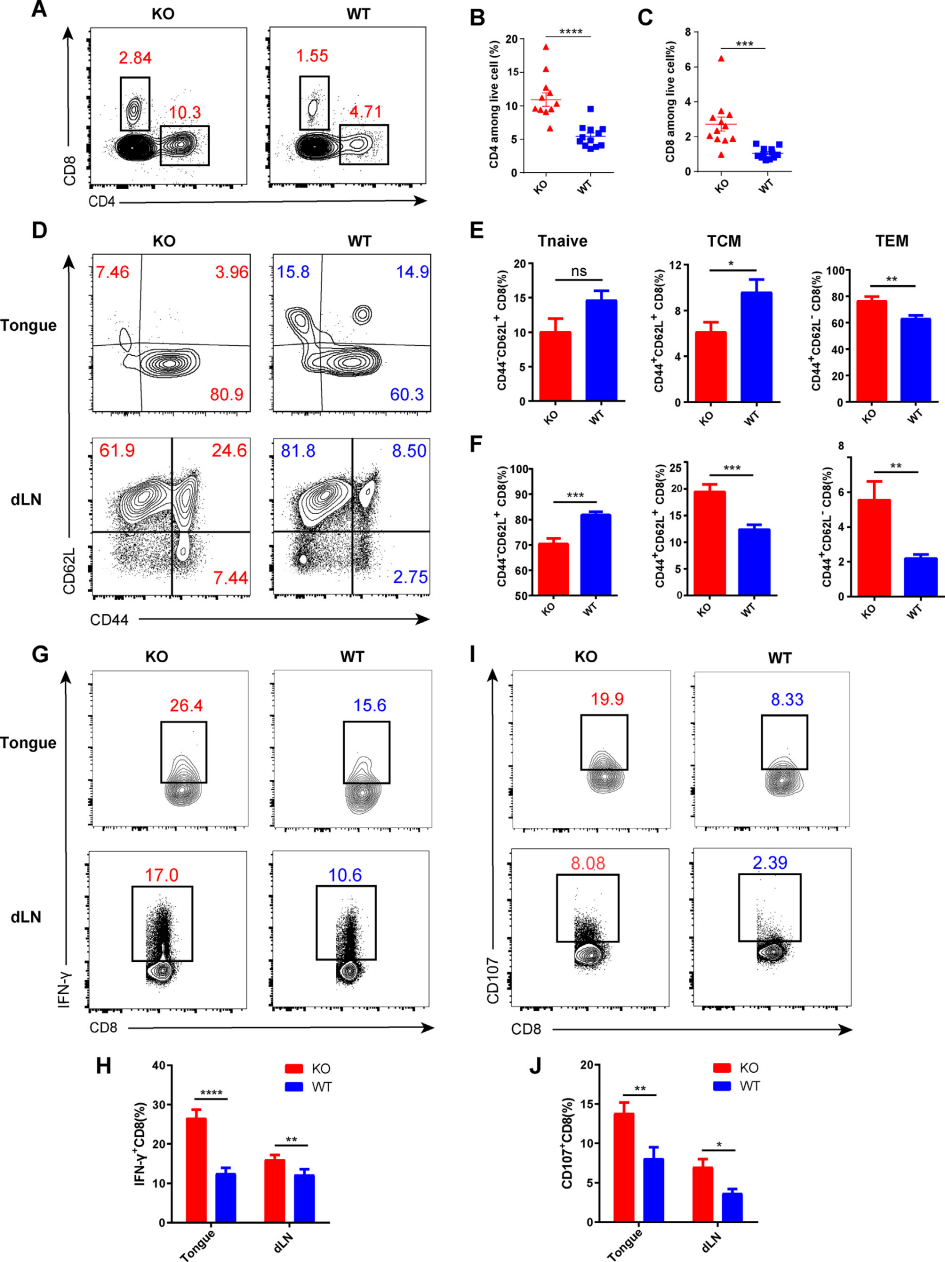
Bcl6 deficiency in Treg cells augments T-cell responses in the tongue immune microenvironment and draining lymph nodes. **(A–C)** The frequency of tongue-infiltrating CD4^+^ and CD8^+^ T cells among live cells. **(D–F)** Activation status of CD8^+^ T cells in the tongue and dLNs assessed by CD44 and CD62L expression. **(G, H)** The proportions of IFN-γ-producing CD8^+^ T cells in the tongue and dLNs. **(I, J)** The proportions of CD107-producing CD8^+^ T cells in the tongue and dLNs. dLNs, draining lymph nodes; Tnaïve, naïve T cells; TCM, central memory T cells; TEM, effector memory T cells. The data were presented as mean ± standard error of the mean and were calculated by unpaired *t*-test; *n* ≥ 3; the data presented are representative of at least three independent experiments; ∗*P* < 0.05, ∗∗*P* < 0.01, ∗∗∗*P* < 0.001, ∗∗∗∗*P* < 0.0001.

T cells, especially CD8^+^ T cells, are essential for immune defense against malignancies. Whereupon, we evaluated the T cell responses in preceding 4NQO-induced HNSCC models using flow cytometry analysis, and the gating strategy was shown in Figure S1. Bcl6 deletion in Treg cells resulted in increased tongue infiltration of T cells, evidenced by the significantly higher percentages of CD4^+^ and CD8^+^ T cells in KO mice (Fig. 2A–C). Then, the frequencies of naïve T cells (CD44^−^ CD62L^+^), central memory T cells (CD44^+^ CD62L^+^), and effector memory T cells (CD44^+^ CD62L^−^) were analyzed. No significant differences in the percentages of tongue-infiltrating CD8^+^ naïve T cells were observed between the two groups while KO mice exhibited a lower frequency of CD8^+^ central memory T cells and a higher frequency of CD8^+^ effector memory T cells compared with WT mice (Fig. 2D, E). Notably, CD8^+^ T cells derived from tumor dLNs of KO mice that exhibited naïve T phenotype were remarkably reduced while CD8^+^ T cells that exhibited central memory T and effector memory T phenotype were significantly increased (Fig. 2D–F). Similarly, KO mice exhibited a higher proportion of tongue-infiltrating non-Treg CD4^+^ effector memory T cells compared with WT mice and there were fewer non-Treg CD4^+^ naïve T cells and more central memory T cells derived from the dLNs of KO mice versus WT mice (Fig. S2A). dLN is the primary site where tumor antigen-specific CD8^+^ T cells are primed and activated by antigen-presenting cells through antigenic peptide-MHC complex. Conventional dendritic cells are the chief antigen-presenting cells that engulf tumor antigens and transport them to the dLNs.^23^^,^^24^ Of note, we detected higher percentages of migratory conventional dendritic cells in the dLNs of KO mice versus WT mice, as well as a significantly higher proportion of migratory CD103^+^ conventional dendritic cells in KO mice which is critical for driving the anti-tumor CD8^+^ T cell responses (Fig. S2B). In addition, CD8^+^ T cells from the dLNs of KO mice exhibited up-regulated expression of proliferation biomarker Ki67 and anti-apoptotic protein Bcl2 compared with WT mice (Fig. S2C). To elucidate whether the better priming and activation of CD8^+^ T cells in the dLNs led to better effector function in KO mice, cytokine production was evaluated. Both the tongue-infiltrating and dLN CD8^+^ T cells derived from KO mice exhibited higher IFN-γ and CD107 production compared with their WT counterparts (Fig. 2G–J). Together, these data suggested that the abrogation of Bcl6 in Treg cells facilitated the priming and activation of CD8^+^ T cells in the dLNs which further infiltrated into the tongue and exhibited better effector function during HNSCC.

### Bcl6 deletion impaired the suppressive function of Treg cells in the dLNs

**Figure 3:**
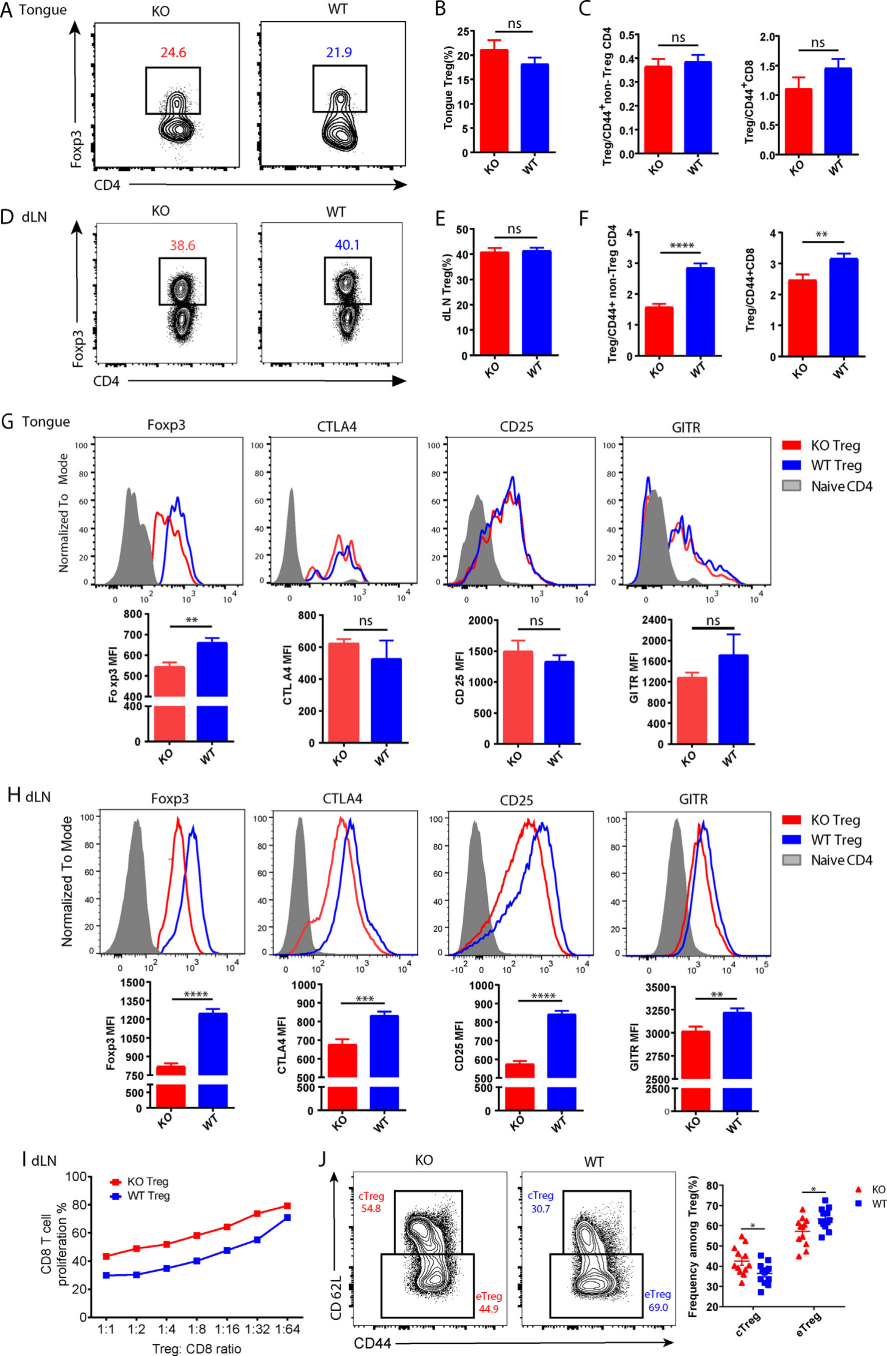
Bcl6 deficiency impairs the suppressive capacity of Treg cells in draining lymph nodes. **(A, B)** The percentages of Treg cells in the tongue. **(C)** The ratio of Treg cells to CD44^+^ non-Treg CD4^+^ T cells and CD44^+^ CD8^+^ T cells in the tongue. **(D, E)** The percentages of Treg cells in the dLNs. **(F)** The ratio of Treg cells to CD44^+^ non-Treg CD4^+^ T cells and CD44^+^ CD8^+^ T cells in the dLNs. **(G, H)** Expression level of Foxp3, CTLA4, CD25, and GITR in tongue-infiltrating Treg cells (G) and dLN Treg cells (H). **(I)***In vitro* suppression assay. CD8^+^ T cell proliferation was assessed after incubation with dLN Treg cells (CD4^+^ CD25^+^ GITR^+^) sorted from 4NQO-treated KO or WT mice in the indicated ratios for 3 days. **(J)** The proportion of cTreg and eTreg in the dLNs. dLNs, draining lymph nodes; cTreg, central Treg cells; eTreg, effector Treg cells. Data were presented as mean ± standard error of the mean and calculated by unpaired *t*-test; *n* ≥ 10; data in each panel are representative of at least three independent experiments; ∗*P* < 0.05, ∗∗*P* < 0.01, ∗∗∗*P* < 0.001, ∗∗∗∗*P* < 0.0001.

To determine whether the improved effector function of CD8^+^ T cells in KO mice resulted from attenuated quantity or quality of Treg cells in HNSCC, we detected the Treg cells in the tongue and dLNs of the 4NQO-induced mice. The percentage of total Treg cells between KO and WT mice was comparable in the tongue (Fig. 3A, B), as well as the ratio of Treg cells to CD44^+^ non-Treg CD4^+^ T cells and CD44^+^ CD8^+^ T cells (Fig. 3C). However, in the dLNs, despite the comparable proportion of total Treg cells (Fig. 3D, E), the ratios of Treg cells to CD44^+^ non-Treg CD4^+^ T cells and CD44^+^ CD8^+^ T cells were remarkably reduced in KO mice versus WT counterparts (Fig. 3F). In addition, several well-recognized factors that mediate the lineage-specificity and suppressive function of Treg cells including Foxp3, CTLA4, CD25, and GITR were examined. Tongue-infiltrating Treg cells derived from KO mice exhibited dramatically reduced expression of Foxp3 compared with WT mice, while the expression of the other three markers showed no differences between these two groups (Fig. 3G). Nevertheless, the expression of these four molecules in KO Treg cells was all significantly repressed compared with WT Treg cells in the dLNs (Fig. 3H). Subsequently, an *in vitro* suppression assay was conducted by co-culturing dLN WT or KO Treg cells isolated from the HNSCC model with naïve prelabeled CD8^+^ T cells in the presence of anti-CD3 and anti-CD28 monoclonal antibodies, and the results revealed that KO Treg cells exhibited inferior suppressive capacity compared with WT Treg cells (Fig. 3I). Treg cells could be divided into central Treg cells and effector Treg cells according to the expression level of CD44 and CD62L. The former was characterized by high expression of CD62L and lower level of CD44 and exhibited less suppressive activity compared with the latter. Notably, we observed a significantly higher proportion of central Treg cells and a lower proportion of effector Treg cells in KO mice versus WT mice (Fig. 3J). Altogether, these results demonstrated that Bcl6 deficiency in Treg cells attenuated the lineage stability and suppressive capacity of Treg cells especially in the dLNs during HNSCC.

### Bcl6 may preserve the lineage stability of Treg cells through the histone H3K4 trimethylation

**Figure 4:**
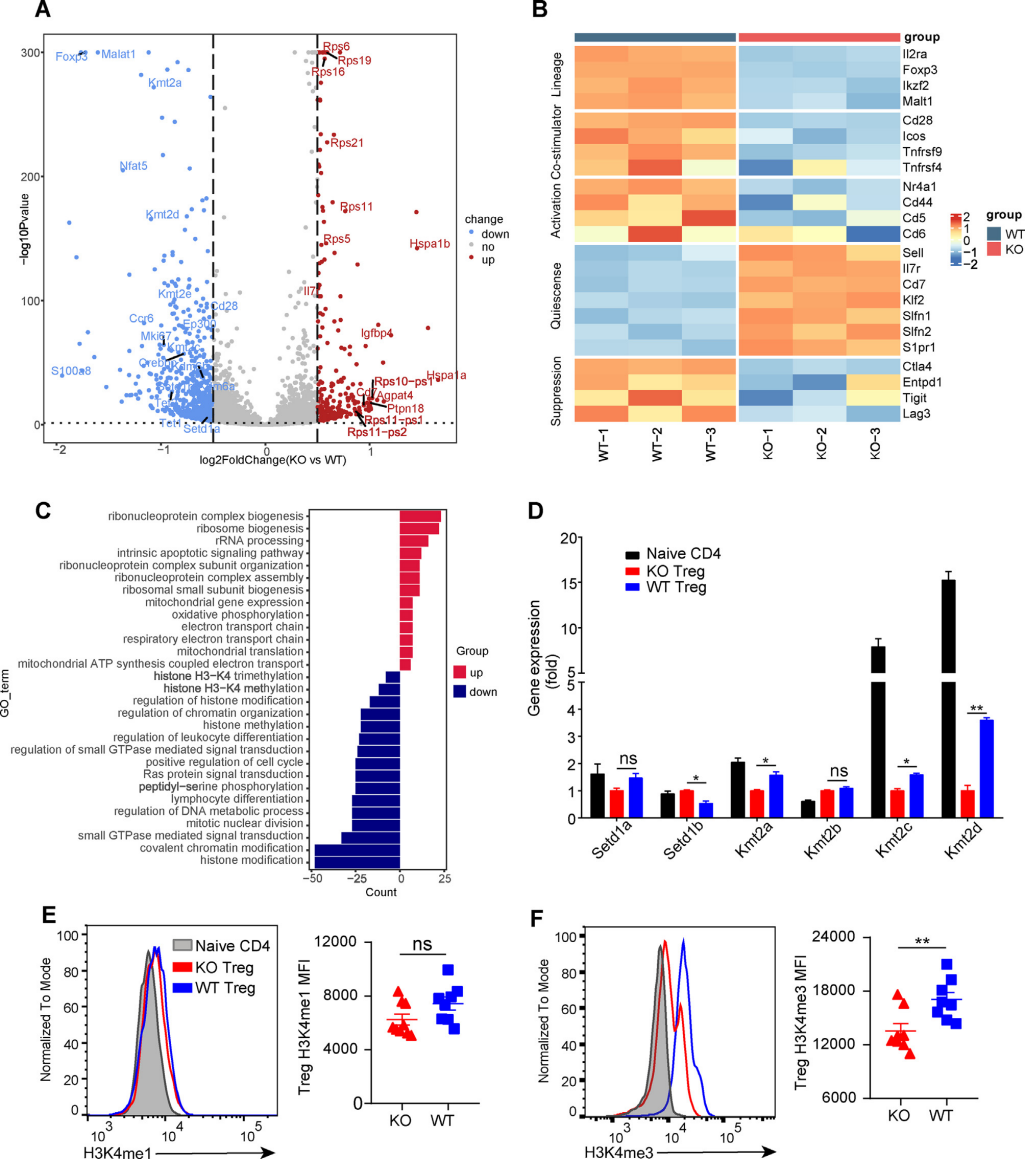
Transcriptional profiles of Treg cells in draining lymph nodes. **(A)** Volcano plots for the differentially expressed genes (DEGs) of KO Treg cells versus WT Treg cells. Red and blue dots denote up-regulated and down-regulated DEGs, respectively, with |log2 fold change| > 0.5 and false discovery rate <0.05. **(B)** Heatmap of selected genes in KO Treg cells and WT Treg cells. **(C)** Gene ontology (GO) pathway analysis of DEGs. Red and blue stripes denote enriched pathways in KO Treg cells and WT Treg cells, respectively. **(D)** Quantitative reverse transcription PCR analysis of the transcription levels of the indicated genes, normalized to their expression in KO Treg cells. **(E,F)** Expression level of H3K4me1 and H3K4m3 in dLN Treg cells. dLNs, draining lymph nodes. Data were presented as mean ± standard error of the mean and calculated by unpaired *t*-test; *n* ≥ 3; ∗*P* < 0.05, ∗∗*P* < 0.01.

To uncover the mechanisms underlying Bcl6 regulating Treg cells in HNSCC, RNA sequencing of WT and KO Treg cells derived from the dLNs in the 4NQO-induced HNSCC model was performed. We identified 382 differentially expressed genes between WT and KO Treg cells, among which *Hspa1a*, *Igfbp4*, *Cd7*, and *Il7r* were found to be up-regulated in KO Treg cells while WT Treg cells exhibited higher expression of *Foxp3*, *Mki67*, *Ccr6*, and histone H3K4 transmethylase genes, including *Setd1a*, *Setd1b*, *Kmt2a*, *Kmt2b*, *Kmt2c*, and *Kmt2d* (Fig. 4A). Notably, we observed down-regulated levels of Treg cell signature genes (*e.g.*, *Il2rα*, *Foxp3*, *Ikzf2*, and *Malt1*) in KO Treg cells versus WT Treg cells (Fig. 4B). In addition, KO Treg cells expressed lower level of costimulatory genes (*e.g.*, *Cd28* and *Icos*), activation-related genes (*e.g.*, *Nr4a1* and *Cd44*), and suppressive function-related genes (*e.g.*, *Ctla4* and *Entpd1*) while higher levels of quiescence-related genes (*e.g.*, *Sell*, *Il7r*, *Cd7*, and *Klf2*) compared with WT Treg cells (Fig. 4B). These results were consistent with the flow cytometry data (Fig. 3D). Gene ontology analysis of differentially expressed genes revealed that genes up-regulated in KO Treg cells were related to ribonucleoprotein complex biogenesis, mitochondrial gene expression, and oxidative phosphorylation. Nevertheless, genes up-regulated in WT Treg cells were enriched in histone modification (especially histone H3K4 methylation which was linked to gene activation), small GTPase-mediated signal transduction, and lymphocyte differentiation (Fig. 4C). Concurrently, quantitative real-time PCR results revealed that KO Treg cells exhibited significantly lower level of *Kmt2a*, *Kmt2c*, and *Kmt2d* compared with WT Treg cells (Fig. 4D). In addition, flow cytometry data indicated that histone H3K4 trimethylation but not the monomethylation was markedly decreased in KO Treg cells versus WT Treg cells (Fig. 4E, F). These data implicated that Bcl6 deficiency down-regulated histone H3K4 trimethylation, which led to reduced expression of Foxp3 and further impaired the lineage stability and suppressive function of Treg cells.

### Combination therapy consisting of Bcl6 inhibitor FX1 and PD-1/PD-L1 ICB repressed the tumor growth of HNSCC

**Figure 5:**
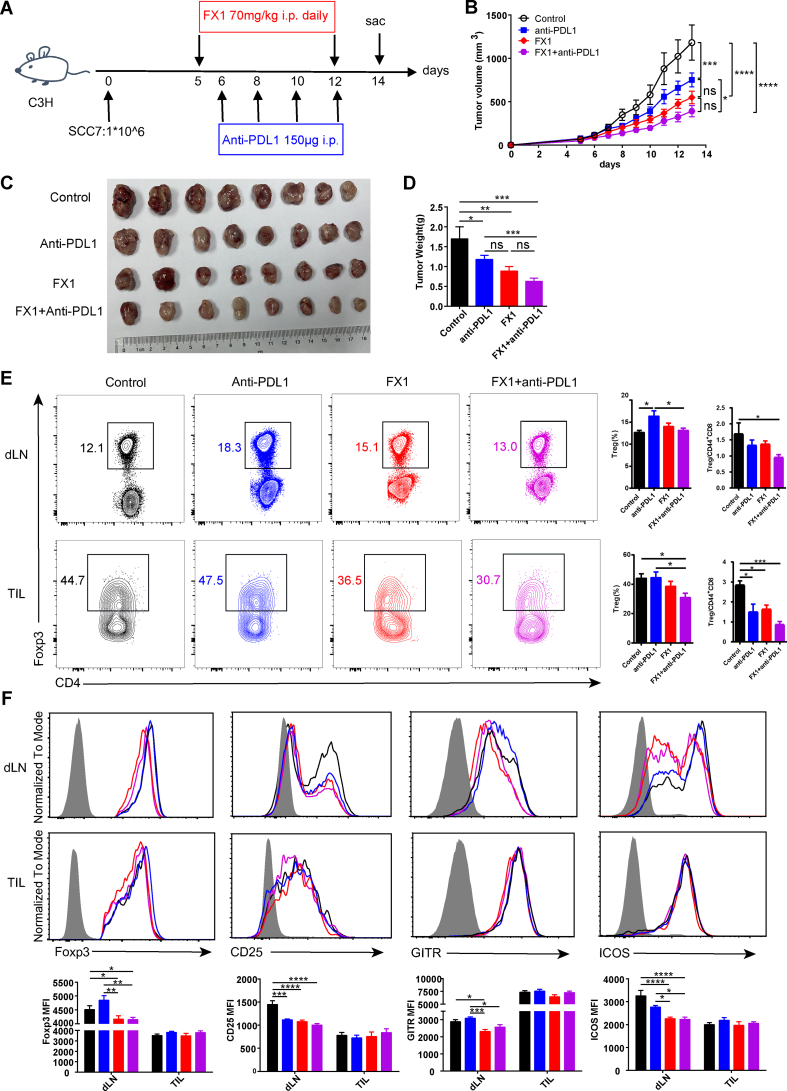
Combination therapy with Bcl6 inhibitor FX1 and anti-PDL1 repressed the tumor growth of murine HNSCC. **(A)** The sketch of the therapy: C3H mice implanted with SCC7 (1 × 10^6^/mouse) on the left flanks were randomly divided into four groups on day 5. Mice in the FX1 group and the combination group were intraperitoneally injected with FX1 (70 mg/kg) daily from day 5 for consecutive 8 days. Mice in the anti-PDL1 group and the combination group were intraperitoneally injected with anti-PDL1 (150 μg) at days 6, 8, 10, and 12. **(B)** Tumor growth curves of treated mice. **(C)** Pictures of tumor samples harvested at day 14. **(D)** Statistical diagram of tumor weight. **(E)** The proportion of Treg cells and the ratio of Treg cells to CD44^+^ CD8^+^ T cells. **(F)** Expression level of Foxp3, CD25, GITR, and ICOS in Treg cells. Data were presented as mean ± standard error of the mean and were calculated by two-way ANOVA (tumor growth curve) or one-way ANOVA; *n* ≥ 8; data in each panel are representative of at least two independent experiments; ∗*P* < 0.05, ∗∗*P* < 0.01, ∗∗∗*P* < 0.001, ∗∗∗∗*P* < 0.0001.

As the deletion of Bcl6 in Treg cells resulted in significantly delayed tumor progression in the 4NQO-induced carcinogenesis model, we hypothesized that Bcl6 may represent a potential therapeutic target in HNSCC treatment. Thus, SCC7 tumor-bearing C3H mice were randomly divided into four treatment groups: i) control; ii) anti-PDL1 antibody; iii) Bcl6 inhibitor FX1, and iv) combination therapy of FX1 and anti-PDL1 (Fig. 5A). Monotherapy with FX1 or anti-PDL1 significantly suppressed the tumor progression. Notably, the combination therapy demonstrated a more pronounced tumor repressive capacity than the anti-PDL1 group (Fig. 5B–D). These data suggested that Bcl6 inhibition significantly enhanced the therapeutic efficacy of anti-PD-L1.

Next, we evaluated the effect of FX1 on Treg cells in HNSCC. Using flow cytometry analysis, we found that the frequency of dLN-derived Treg cells in the FX1 treatment group and combination treatment group exhibited no significant differences compared with the control group, while the proportion of Treg cells in the combination treatment group was significantly lower than that in the anti-PD-L1 group. In addition, the proportion of tumor-infiltrating Treg cells in the combination treatment group was significantly lower than that in the control group and the anti-PD-L1 group (Fig. 5E). Importantly, the ratios of Treg cells to CD44^+^ CD8^+^ T cells both in the dLNs and tumor-infiltrating lymphocytes were remarkably reduced in the combination treatment group (Fig. 5E). FX1 administration reduced the expression of Foxp3, CD25, GITR, and ICOS in Treg cells from the dLNs, but had little impact on the expression of these molecules in tumor-infiltrating Treg cells (Fig. 5F). Correspondingly, the proliferation of CD8^+^ T cells from both the dLNs and tumor-infiltrating lymphocytes in the combination treatment group was enhanced compared with the control group, indicated by increased expression of Ki67 (Fig. S3A). Simultaneously, compared with the control group, the combination treatment group exhibited a significantly higher frequency of TNF-α^+^ IFN-γ^+^ CD8^+^ T cells in the dLNs (Fig. S3B), as well as increased proportion and absolute number of IFN-γ^+^ and GZMB^+^ CD8^+^ T cells in tumor-infiltrating lymphocytes (Fig. S3C, D). Collectively, these data suggested that Bcl6 is a suitable therapeutic target in HNSCC. Bcl6 inhibitor FX1 significantly delayed the tumor growth of HNSCC which further enhanced the anti-tumor efficacy of PD-1/PD-L1 ICB.

## Discussion

Treg cells are a sizable proportion of T cells that contribute to the immunosuppressive tumor microenvironment in HNSCC, of which the functional state and infiltration within the tumor microenvironment are closely related to the clinical outcome of cancer patients. Using the 4NQO-induced murine multistage carcinogenesis model, we previously found that Treg cells accumulated in the tumor microenvironment and dLNs over the OSCC development which related to resistance to ICB therapy.^25^ Bcl6 is a well-recognized decisive factor in T follicular helper and T follicular regulatory cell differentiation. However, the role of Bcl6 in Treg cells especially in terms of HNSCC has not been reported. In this study, we established a 4NQO-induced HNSCC model in KO (Treg cell-restricted deletion of Bcl6) mice and found that the malignant transformation was significantly delayed in KO mice, evidenced by smaller and fewer tongue lesions compared with their WT counterparts. Bcl6 deficiency in Treg cells led to increased infiltration of T cells in the tongue along with better effector functions, which may be attributed to the better priming and activation of T cells in the dLNs. In addition, the combination therapy with Bcl6 inhibitor FX1 and PD-1/PD-L1 ICB significantly repressed the tumor growth of murine HNSCC.

The differentiation and function of Treg cells depend on its lineage-specifying transcription factor Foxp3, which is critical for Treg cell-mediated immune homeostasis.^26^, ^27^, ^28^ In the 4NQO-induced HNSCC model, despite the comparable proportion of Treg cells, the deletion of Bcl6 lead to extensively repressed Foxp3 expression in Treg cells from the tongue and dLNs, indicating the pivotal role of Bcl6 in maintaining the lineage stability of Treg cells. Besides, based on the activation status, Treg cells can be subdivided into central Treg cells (also called resting Treg cells) and effector Treg cells (also called activated Treg cells).^29^ Central Treg cells are characterized by CCR7^high^ CD62L^high^ CD44^low^ phenotypes with less suppressive activity when compared with effector Treg cells, and these central Treg cells are primarily localized in secondary lymphoid tissues. Emerging evidence supports that effector Treg cells derive from central Treg cells and express higher levels of effector markers, for instance, CD44, CTLA4, GITR, and ICOS. These effector Treg cells dominate non-lymphoid peripheral tissues such as mucosa, lung, and fat tissue.^30^^,^^31^ Coincident with that, we found that the majority of Treg cells in the tongue were effector Treg cells in our 4NQO-induced carcinogenesis model. Furthermore, the Bcl6 deletion in Treg cells resulted in decreased effector Treg cells and increased central Treg cells in the dLN which provide an appropriate milieu for better CD8^+^ T cell priming and activation. Nevertheless, the authentic connection between central Treg cells and effector Treg cells has not been completely demonstrated and more studies are needed to fully elucidate the role of Bcl6 on these cells.

Consistent with the results from flow cytometry, RNA sequencing data revealed that the transcription levels of Treg cell lineage and suppressive function-related genes (*Foxp3*, *Il2rα*, *Cd28*, *etc*.) in KO Treg cells were significantly disrupted compared with WT Treg cells. A previous study showed that Treg cells from both the tumor microenvironment and dLNs in HNSCC patients exhibited a CCR6^+^ effector Treg cell phenotype, and CCR6^+^ Treg cells expressed a higher level of FOXP3 than CCR6^−^ Treg cells, which indicated a stronger suppressive function.^32^ In the 4NQO-induced HNSCC model, we found that KO Treg cells exhibited a significantly lower level of *Ccr6* compared with WT Treg cells, which was consistent with the lower effector Treg cell phenotype of KO Treg cells.

Histone methylation exhibits an intricate effect on the gene expression. For instance, the monomethylation of H3K27 and H3K9 is linked to gene activation, whereas their trimethylation is linked to gene repression. As for H3K4, both monomethylation and trimethylation are linked to gene activation.^33^^,^^34^ Our gene ontology pathway analysis of differentially expressed genes demonstrated that the down-regulated genes in KO Treg cells versus WT Treg cells were significantly enriched in the histone H3K4 methylation pathway. Quantitative reverse transcription PCR results also uncovered that H3K4 transmethylase genes, including *Kmt2a*, *Kmt2c*, and *Kmt2d*, were remarkably decreased in KO Treg cells versus WT Treg cells. Indeed, Kmt2d has been previously reported to promote Treg cell development by establishing the enhancer landscape.^35^ In addition, flow cytometry data indicated that H3K4me3 but not H3K4me1 was dramatically down-regulated in Treg cells in the absence of Bcl6. Notably, BCL6 binding sites relative to the H3K4me3 in human B-cell lymphoma cells and human follicular helper T cells have been previously identified through chromatin immunoprecipitation sequencing.^36^^,^^37^ However, how Bcl6 regulates the H3K4 trimethylation in Treg cells during HNSCC requires further investigation.

To avoid the massive systemic inflammation caused by the full repression of Bcl6, the majority of the published Bcl6 inhibitors were developed to disrupt the interaction between the Bcl6 BTB domain and its corepressors.^38^^,^^39^ FX1 is the first reported Bcl6 inhibitor endowed with higher affinity for the Bcl6 BTB domain than the endogenous corepressors and exhibited a favorable pharmacokinetic *in vivo* and lack of toxicity, inflammation, or infection.^40^ To date, FX1 has been reported to exhibit therapeutic effects on the experimental B-cell lymphoma,^40^^,^^41^ lymphoblastic leukemia,^42^^,^^43^ and autoimmune disease,^44^ while the application of FX1 in the therapy of solid tumors has not been reported. In the present study, we proved that FX1 suppressed the tumor growth of murine HNSCC and enhanced the anti-tumor efficacy of anti-PD-L1. Consistent with the results in KO mice, FX1 administration reduced the expression of Foxp3, CD25, GITR, and ICOS in Treg cells from the dLNs. Importantly, the ratios of Treg cells to CD44^+^ CD8^+^ T cells in both the dLNs and tumor-infiltrating lymphocytes were remarkably reduced in the FX1-treated and combination treatment group, which suggested that the therapeutic effects of FX1 are largely mediated by Treg cells. Of note, other immune cells, stromal cells, or tumor cells expressing Bcl6 could be also targeted by FX1.^45^ In the present study, SCC7 cells exhibited a similar level of Bcl6 compared with normal mouse oral epithelial cells (data not shown), which excluded the effect of FX1 on the tumor cell. However, the underlying mechanisms need to be thoroughly explored in the future.

Collectively, our results reveal that Bcl6 plays a pivotal role in maintaining the lineage stability and suppressive function of Treg cells during HNSCC. Bcl6 deficiency in Treg cells substantially delayed the malignant transformation in the 4NQO-induced HNSCC model. Targeting Bcl6 repressed the tumor growth of murine HNSCC which further enhanced the therapeutic efficacy of ICB therapy, indicating that Bcl6 inhibition represents a promising strategy for clinical HNSCC treatment.

## Funding

This project was supported by grants from the National Science Foundation for Outstanding Young Scholars of China (No. 82322031 to Q.H.; 82122028 to L.X.), the National Natural Science Foundation of China (No. 82173094 to L.X.; 82372837 to Z.C.), the National Science Foundation for Outstanding Young Scholars of Chongqing, China (No. CSTB2024NSCQ-JQX0008 to Q.H.; CSTB2022NSCQ-JQX0015 to L.X.), the Natural Science Foundation of Chongqing Municipality, China (No. CSTB2023NSCQ-LZX0010 to Q.H.; CSTB2023NSCQ-BHX0101 to S.W.; CSTB2023NSCQ-BHX0102 to Z.O.), and the Natural Science Foundation of Guang Dong, China (No. 2024A1515010375 to Q. H.).

## CRediT authorship contribution statement

**Shuqiong Wen:** Conceptualization, Data curation, Formal analysis, Funding acquisition, Investigation, Methodology, Project administration, Writing – original draft, Writing – review & editing. **Xingxing Su:** Conceptualization, Data curation, Formal analysis, Investigation, Validation, Writing – original draft, Writing – review & editing. **Junyi Guo:** Data curation, Formal analysis, Project administration, Software, Visualization. **Zhanpeng Ou:** Funding acquisition, Investigation, Project administration, Supervision, Validation. **Lisha Wang:** Data curation, Project administration, Validation, Visualization. **Zhengliang Yue:** Methodology, Validation, Visualization. **Jing Zhao:** Methodology, Software, Validation. **Ling Ran:** Investigation, Project administration, Visualization. **Jianjun Hu:** Investigation, Software, Visualization. **Yuzhu Wang:** Project administration, Supervision, Validation. **Mengqu Ran:** Investigation, Methodology, Supervision. **Qinyi He:** Project administration, Validation. **Ping Ji:** Conceptualization, Supervision, Writing – review & editing. **Lilin Ye:** Conceptualization, Methodology, Supervision. **Zhiyu Chen:** Conceptualization, Funding acquisition, Methodology, Supervision, Writing – review & editing. **Lifan Xu:** Conceptualization, Methodology, Resources, Supervision, Writing – original draft, Writing – review & editing, Funding acquisition. **Qizhao Huang:** Conceptualization, Funding acquisition, Methodology, Resources, Supervision, Writing – original draft, Writing – review & editing.

## Data availability

RNA sequencing gene expression data described in Fig. 4A–C has been deposited at the Sequence Read Archive (SRA) with accession numbers PRJNA911002.

## Conflict of interests

The authors declared no conflict of interests.
