# Supplementary material for: Bcl6 controls the stability and suppressive function of regulatory T cells in head and neck squamous cell carcinoma
Source: Genes Dis. 2024 Dec 26;12(4):101505. doi: 10.1016/j.gendis.2024.101505 (PMC12033904; doi:10.1016/j.gendis.2024.101505)
Supplement: Multimedia component 1 [file mmc1.docx]

**Supplementary Material**


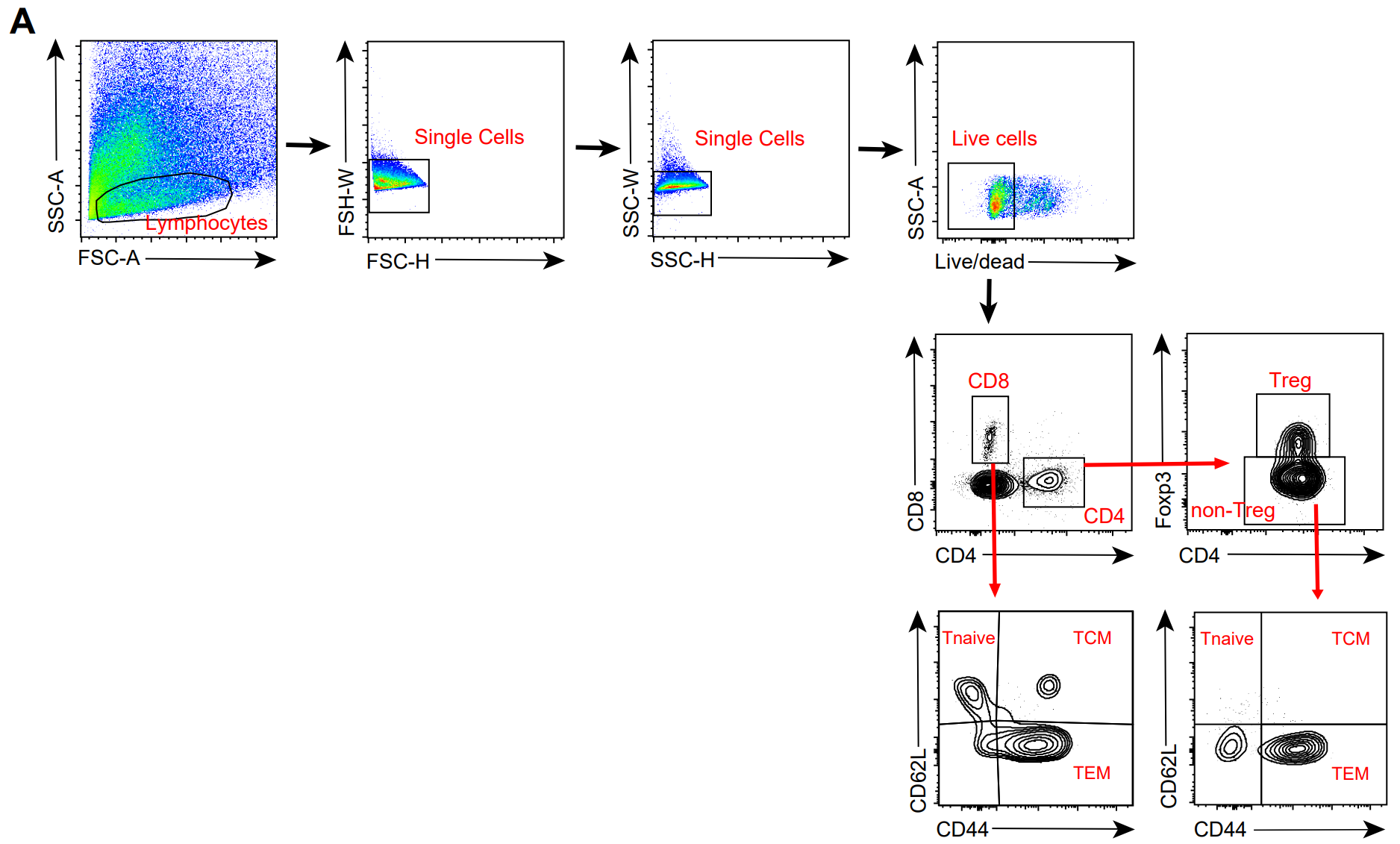


**Figure S1** Flow cytometry gating strategy.


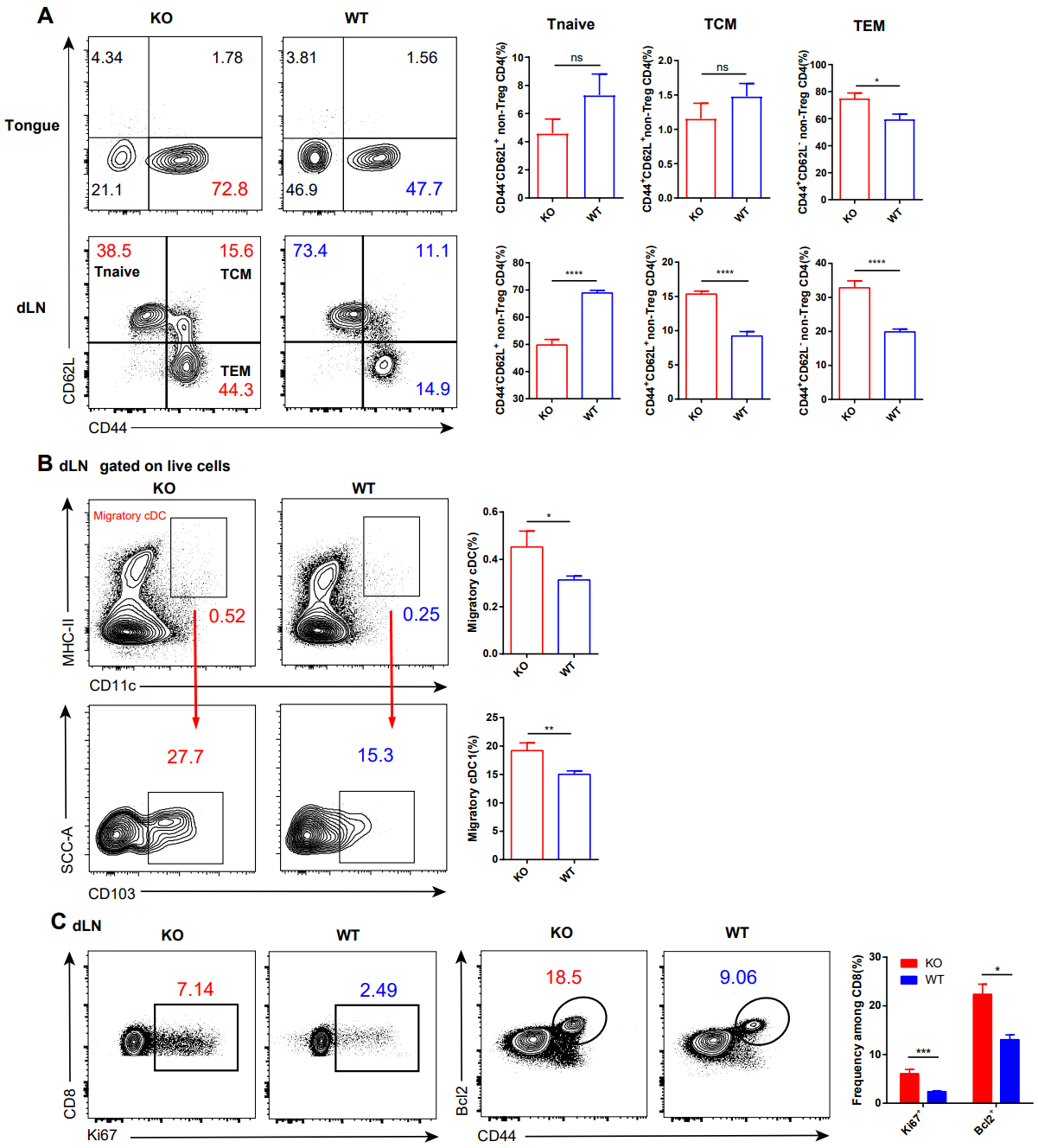


**Figure S2** **(A)** Activation status of CD4^+^T cells in the tongue and dLN assessed by CD44 and CD62L expression. **(B)** The proportion of migratory cDC and CD103^+^ migratory cDC1 in dLN. **(C)** The frequency of Ki67^+^CD8^+^ and Bcl2^+^CD8^+^T cells in dLN. Data presented as mean ±SEM. Unpaired *t* test. **p*< 0.05, ***p* < 0.01, *****p* < 0.0001. (Related to Figure2)


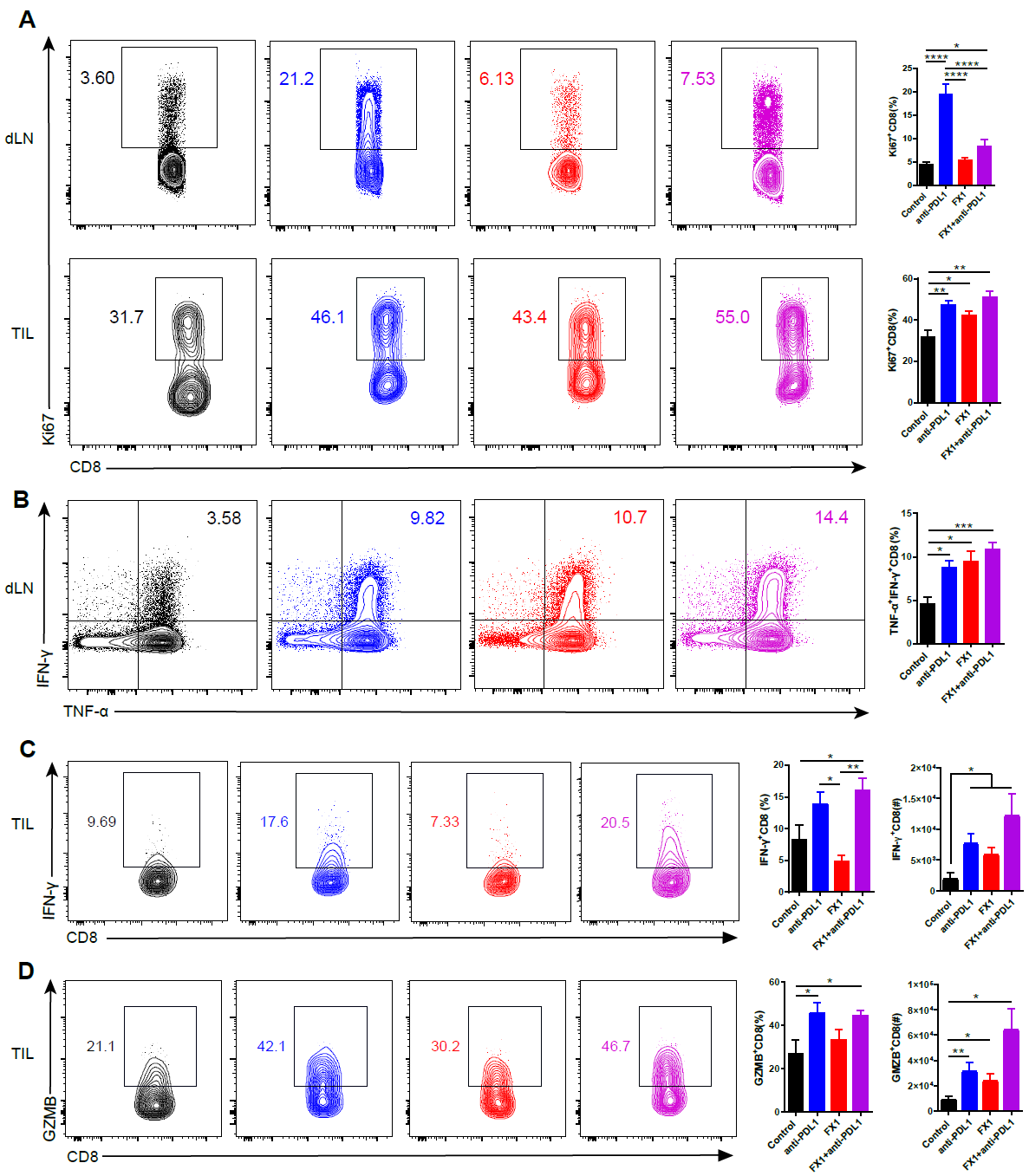


**Figure S3** **(A)** The proportion of Ki67^+^CD8^+^T cells in dLN and TIL. **(B)** The proportion of TNF-α^+^IFN-γ^+^CD8^+^T cells in dLN. **(C)** The proportion and absolute number of IFN-γ^+^CD8^+^T cells in TIL. **(D)** The proportion and absolute number of GZMB^+^CD8^+^T cells in TIL. Data presented as mean ±SEM. one-way ANOVA. **p* < 0.05, ***p* < 0.01, ****p* < 0.001, *****p* < 0.0001. (Related to Figure5)

**Table S1. Anti-mouse antibodies and reagents for flow cytometry.**

| **Antibody target/Reagent** | **Clone** | **Source** |
| --- | --- | --- |
| CD4-Brilliant Violet 510 | RM4-5 | Biolegend |
| CD8-PerCP/Cyanine5.5 | 53-6.7 | Biolegend |
| CD8-Brilliant Violet 605 | 53-6.7 | Biolegend |
| CD44-PerCP | IM7 | eBioscience |
| CD44-Alexa Fluor700 | IM7 | Biolegend |
| CD62L-APC | MEL-14 | Biolegend |
| CD69-FITC | H1.2F3 | Biolegend |
| CD69-PE/Cyanine7 | H1.2F3 | Biolegend |
| CD103-PE | 2E7 | Biolegend |
| CD103-Brilliant Violet 421 | 2E7 | Biolegend |
| Live/Dead Kit | L10119 | Life Technologies |
| MHC-Ⅱ- PE/Cyanine7 | AF6-120.1 | Biolegend |
| CD11c-PerCP | N418 | Biolegend |
| Ki67-FITC | B56 | BD Biosciences |
| Bcl2-FITC | 7/Bcl-2 | BD Biosciences |
| IFNγ-Brilliant Violet 421 | XMG1.2 | Biolegend |
| TNFα-PE/Cyanine7 | MP6-XT22 | BD Biosciences |
| CD107a-FITC | 1D48 | BD Biosciences |
| CD107b-FITC | ABL-93 | BD Biosciences |
| Foxp3-APC | FJK-16s | Biolegend |
| Foxp3-PE | FJK-16s | Biolegend |
| CTLA4-PE | UC10-4B9 | Biolegend |
| CD25-APC | PC61.5 | eBioscience |
| GITR-PE/Cyanine7 | DTA-1 | eBioscience |
| H3K4me1 | D1A9 | Cell signaling technology |
| H3K4me3 | C42D8 | Cell signaling technology |
| donkey anti-rabbit IgG-Alexa Fluor® 488 |  | invitrogen |

**Table S2. Primers used in quantitative PCR.**

| **Gene symbol** | **Forward primer** | **Reverse primer** |
| --- | --- | --- |
| ***Actb*** | AATCGTGCGTGACATCAAAG | GGATTCCATACCCAAGAAGG |
| ***Setd1a*** | TTCCAGTGGCGGAACTACAAG | AACCGGGAGGGAAAAGTCTCT |
| ***Setd1b*** | CTCACCTGAACCCTCACCAC | GTTTCTCCCTCATGTCCGCA |
| ***Kmt2a*** | ATGAGCAGTTCTTAGGTTTTGGC | CTCCCGCGAGGTTTTCGAG |
| ***Kmt2b*** | GTGTCCTTTGCTGCCACTTG | CCTCGCCTGCTGAATAGTGA |
| ***Kmt2c*** | GGTCTCTGTTGGCGTAGAAGC | AAGGTAAGTCCAGGTGTTACTCT |
| ***Kmt2d*** | AATGCAAAGTGTGCCAATCGT | CCATAGGCGGTTTTAAGCAGA |
